# Myostatin Is Upregulated Following Stress in an Erk-Dependent Manner and Negatively Regulates Cardiomyocyte Growth in Culture and in a Mouse Model

**DOI:** 10.1371/journal.pone.0010230

**Published:** 2010-04-19

**Authors:** Lawrence T. Bish, Kevin J. Morine, Meg M. Sleeper, H. Lee Sweeney

**Affiliations:** 1 Department of Physiology, University of Pennsylvania School of Medicine, Philadelphia, Pennsylvania, United States of America; 2 Section of Cardiology, Department of Clinical Studies, Veterinary Hospital of the University of Pennsylvania, Philadelphia, Pennsylvania, United States of America; Institute of Evolutionary Biology (CSIC-UPF), Spain

## Abstract

Myostatin is well established as a negative regulator of skeletal muscle growth, but its role in the heart is controversial. Our goal in this study was to characterize myostatin regulation following cardiomyocyte stress and to examine the role of myostatin in the regulation of cardiomyocyte size. Neonatal cardiomyocytes were cultured and stressed with phenylephrine. Adenovirus was used to overexpress myostatin or dominant negative myostatin in culture. Adeno-associated virus was used to overexpress myostatin or dominant negative myostatin in mice. Myostatin is upregulated following cardiomyocyte stress in an Erk-dependent manner that is associated with increased nuclear translocation and DNA binding activity of MEF-2. Myostatin overexpression leads to decreased and myostatin inhibition to increased cardiac growth both in vitro and in vivo due to modulation of Akt and NFAT3 pathways. Myostatin is a negative regulator of cardiac growth, and further studies are warranted to investigate the role of myostatin in the healthy and failing heart.

## Introduction

Myostatin, a member of the TGF-β family, is a well-established negative regulator of skeletal muscle mass. Myostatin is synthesized as a 376 amino acid pre-propeptide [1]. Once the 24 amino acid signal sequence is cleaved, the remaining full length myostatin contains a 242 amino acid propeptide domain and a 110 amino acid C-terminus domain. The propeptide domain is inhibitory, while the C-terminal domain is the active region of the peptide. The C-terminal domain contains 9 cysteine residues critical for dimerization, and it is this homodimer that is the active protein.

Myostatin undergoes extensive post-translational modification. After translation, disulfide bonds form in both the propeptide and C-terminal regions to create a homodimer. In vitro evidence suggests that it is then cleaved by a paired dibasic amino acid cleavage enzyme (PACE)/furin serine protease at its RSRR (aa 263–266) sequence to produce an N-terminal propeptide region and a C-terminal region [2]. The propeptide continues to associate non-covalently with the C-dimer, locking it in an inactive form. This inactive, or latent, complex, composed of propeptide and C-dimer, is then secreted into circulation. In vitro evidence suggests that BMP-1/tolloid matrix metalloproteases can cleave the propeptide at D76 [3]. Once cleaved, the propeptide degrades, and the active C-dimer is released to bind its receptor, which initiates intracellular Smad phosphorylation and activation [4]. Recent evidence also suggests that full-length myostatin can be secreted and activated locally in the extracellular matrix [5].

The myostatin knockout mouse exhibits a 2–3 fold increase in skeletal muscle mass due to a combination of hyperplasia and hypertrophy [6]. This phenotype has led to the widely accepted conclusion that myostatin acts as a chalone, a negative growth regulator secreted by the tissue on which it acts, to inhibit skeletal muscle growth. This conclusion has been supported by several other studies in normal [7], [8], [9], [10], [11], [12], [13], [14], [15] and dystrophic mice [16], [17], [18], [19], [20], [21] as well as by the double-muscled Belgian Blue and Piedmontese cattle breeds [22], which harbor a naturally occurring mutation in the myostatin gene. Mutations leading to double muscling have also been reported in Texel sheep and whippets [23]. In addition, a human case has been reported in which a splicing mutation in the myostatin gene has led to increased muscle mass [24].

These previous studies all describe the role of myostatin in skeletal muscle. Sharma et al. was the first group to report myostatin expression in the heart using both RT-PCR and Western blot [25]. In addition, in a sheep model of myocardial infarction, the same group demonstrated that myostatin protein is upregulated for up to one month following infarct with a peak occurring at one week. In humans, it has also been reported that myostatin activation is increased in the myocardium of patients in heart failure and that circulating myostatin is increased in the serum of patients in heart failure compared to healthy controls [26].

In vitro, it was found that myostatin is upregulated following the cyclic stretch of cardiomyocytes. Shyu et al. have demonstrated that IGF-1, myostatin, and p38 phosphorylation increase subsequent to cyclic stretch of cardiomyocytes [27]. Furthermore, myostatin did not increase if IGF-1 and/or p38 signaling was blocked before the stretch stimulus. This suggests that following a hypertrophic stimulus, IGF-1 is secreted to stimulate cell growth. Thereafter, however, myostatin may be secreted to negatively regulate this growth as part of a feedback loop.

Recently, several studies have examined the role of myostatin in cardiac growth and hypertrophy. Myostatin expression seems to be dynamically regulated during embryonic and neonatal development such that low expression corresponds with a high proliferative index [28]. In addition, cardiac expression of myostatin is upregulated during both physiologic hypertrophy [29] and pathologic hypertrophy [30] in the rat, supporting the involvement of myostatin in a negative feedback loop that controls cardiac size. However, manipulation of myostatin expression in transgenic and knockout mice has generated conflicting results. Although investigators seem to agree that myostatin transgenic mice have smaller hearts [31], [32], myostatin knockout mice have been reported to have larger hearts [32], smaller hearts [33], smaller hearts that exhibit an increased hypertrophic response [34], or normal hearts [35].

As a result, the role of myostatin in the regulation of cardiac size remains unclear. This information is especially important in light of the ongoing clinical trials using myostatin inhibition via an antibody approach as a potential treatment for several types of muscular dystrophy [36]. Since many of these patients can be expected to experience some form of cardiomyopathy in the future, it is important to discern how myostatin inhibition will impact the injured heart.

With this clinical consideration in mind, the present study was undertaken to investigate the regulation and function of myostatin in the heart. In the first arm of the study, we used phenylephrine (PE) to stress neonatal cardiomyocytes in culture. PE, an α-agonist, has been previously used as a cardiac stressor to evaluate myostatin signaling in the heart [34]. Our goals were 1.) to identify growth pathways activated by PE stress, 2.) to quantify myostatin activation following cardiac stress, and 3.) to use small molecule inhibitors of each activated growth pathway to identify possible upstream regulators of myostatin. Based on data from the previous cardiac report and from others in skeletal muscle, we chose to evaluate the following growth pathways: Akt and its downstream target GSK-3β, Erk, and NFAT3 [37], [38], [39], [40]. We also investigated the role of the transcription factor MEF-2 in myostatin regulation based on data reporting that the myostatin promoter contains a MEF-2 binding domain [41]. Akt, GSK-3β, and Erk, and MEF-2 are activated by phosphorylation, while NFAT is activated by dephosphorylation.

In the second arm of the study, we used adenovirus in culture to manipulate myostatin expression both positively and negatively and examined the effect on cell size, number, and activation of the same key growth pathways. Finally, we used adeno-associated virus in mice to manipulate myostatin expression both positively and negatively and examined the effect on cardiac size, function, and signaling through the same key growth pathways.

## Results

### Effect of phenylephrine (PE) on growth pathway activation, cell size, and cell number in neonatal cardiomyocytes (NCM)

**Figure 1:**
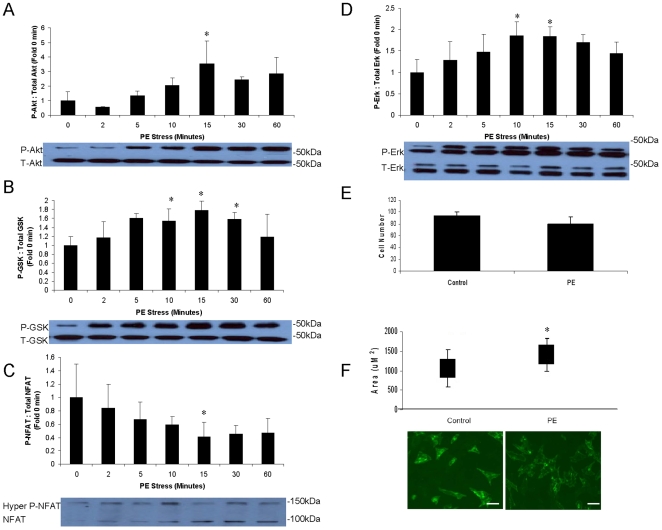
PE induces activation of growth and stress pathways and leads to increased growth in NCM's. Cardiomyocytes were isolated from neonatal rats and grown to confluency. Cells were then stressed with PE (100 uM) or fresh media only (control) for up to 60 minutes to assess activation of growth and stress pathways. Cells were stressed for 18 hours for assessment of growth and proliferation. Each condition was tested n = 4 times. *p<0.05 vs. 0 min (ANOVA). Phospho-specific Western blots were performed to probe the activation states of (A) Akt (60 kDa) (B) GSK3-β (46 kDa) (C) NFAT3 (ranges from approximately 90–140 kDa, depending on phosphorylation state, [59]) and (D) Erk (42 and 44 kDa). For Akt, GSK3-β, and Erk, phosphorylation does not induce a significant increase in molecular weight; therefore, Western blots were probed with the phospho-specific antibody, stripped, and reprobed for total protein. Both blots are shown below each graph. (P =  phospho, T = total, for each protein.) For NFAT, phosphorylation does induce a significant change in molecular weight and gel shift, so only one Western blot is demonstrated for this protein. (E) Cell number was counted in 3 fields for each treatment, and (F) cell surface area was quantified in 3 fields using Fovea Pro software after laminin staining. Approximately 500–800 cells were counted per condition. The plot represents mean ± standard deviation. Representative NCM's 18 hrs following PE stress or control are displayed. Scale bar represents 200 µm. Magnification is 40X.

**Figure 2:**
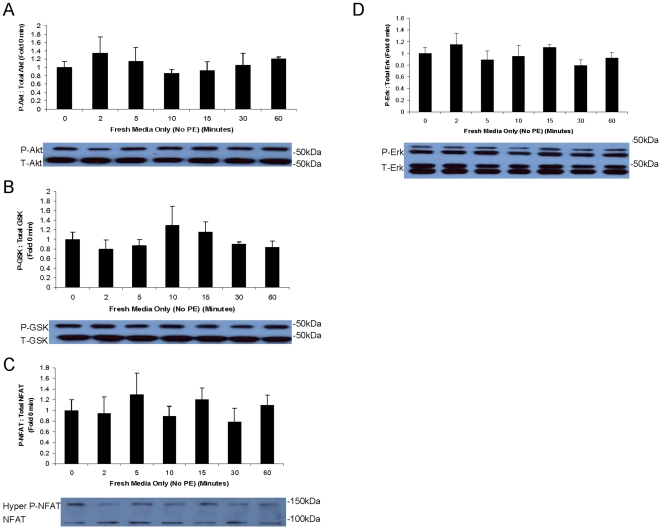
Control Phospho-Western blots. Cardiomyocytes were isolated from neonatal rats and grown to confluency. These control NCM's were grown alongside the NCM's undergoing PE stress but received fresh media only (no PE) during the 60 minute time course. Phospho-specific Western blots were performed to assess the activation states of (A) Akt, (B) GSK3-β, (C) NFAT3, and (D) Erk. No significant differences were observed following the addition of fresh media without PE. Each condition was tested n = 4 times.

PE is a cardiac stressor, and it was hypothesized that PE stress will activate important growth and stress pathways in the heart, including Akt, NFAT3, and Erk. This will be followed by an increase in cell size and possibly number. To test this hypothesis, NCM's were stressed with PE over a time course of 0–60 minutes to examine activation of signaling pathways. Cell size and number were assessed after 18 hours of PE stress. As shown in Figures 1A–D, PE stress did indeed activate Akt (and downstream GSK3- β), NFAT3, and Erk by 15 minutes. (NFAT is activated by dephosphorylation, while the others are activated by phosphorylation.) PE had no effect on cell number (Fig. 1E), but induced an approximately 20% increase in cell size (Fig. 1F). Western blots from control cells which received fresh media only (no PE) during the 60 minute time course revealed that media alone did not induce significant activation of the growth pathways examined (Fig. 2A–D). These data establish that PE is an effective cardiac stressor.

### Effect of PE on myostatin expression and activation in NCM's

**Figure 3:**
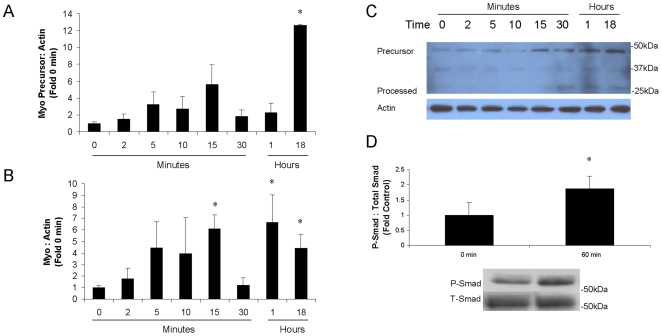
PE increases myostatin translation and activation in NCM's. Cardiomyocytes were isolated from neonatal rats and grown to confluency. Cells were then stressed with PE (100 uM) or media only (control) for up to 18 hours. Each condition was tested n = 4 times. *p<0.05 vs. 0 min (ANOVA). Western blot was performed using the C-terminal antibody to assess levels of (A) myostatin precursor and (B) active myostatin. (C) A representative myostatin blot is depicted. Predicted molecular weights for the various forms of myostatin are as follows: full length precursor monomer (43–45 kDa); propeptide (30–32 kDa); C-terminus monomer (12–13 kDa); active C-dimer (24–26 kDa). Glycosylation may cause these forms to run approximately 5–10 kDa higher than predicted on PAGE. (D) Phospho-specific Western blot was performed to assess activation of Smad (58 kDa).

It was hypothesized that myostatin expression and activation are increased during the hypertrophic response in the heart in an effort to exert negative feedback on cardiac growth. To test this hypothesis, the hypertrophic response was modeled in vitro by treating cultured NCM's with PE as above, and levels of myostatin precursor protein and activated myostatin were analyzed. As shown in Figure 3A, there was no significant increase in myostatin precursor protein from 0–60 minutes, but precursor was increased by 18 hours. Myostatin activation was increased by 60 minutes, and this effect was sustained through 18 hours (Fig. 3B). A representative Western blot is shown in Figure 3C. Since myostatin signals through the Smad pathway, the increased Smad phosphorylation that was observed at 60 minutes confirms the increase in myostatin activation (Figure 3D). These data indicate that myostatin is upregulated following PE stress.

### Myostatin regulation in NCM's

**Figure 4:**
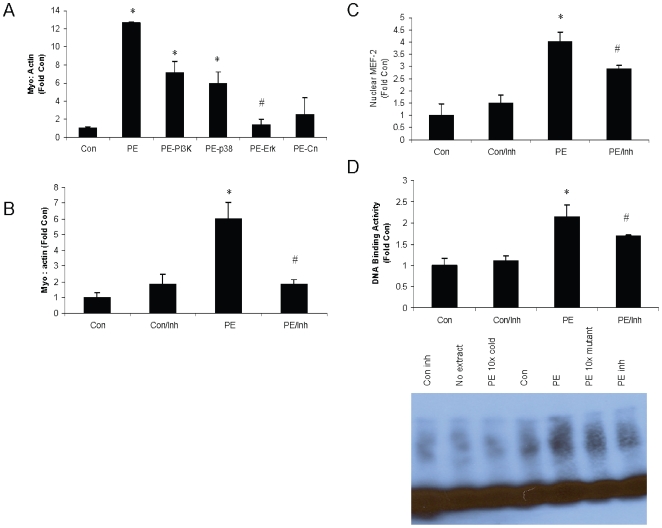
Myostatin upregulation following PE stress is Erk dependent via MEF-2. Cardiomyocytes were isolated from neonatal rats and grown to confluency. Cells were treated with small molecule inhibitors of PI3K (wortmannin, 5 nM), p38 (SB203580, 3 uM), Erk (PD98059, 50 uM), calcineurin (cyclosporine, 1 uM), or control 1 hour prior to 18 hours of PE stress (100 uM) and compared to baseline (no PE, no inhibition). Each condition was tested n = 4 times. (A) Western blot was performed to assess expression of the myostatin precursor protein using the C-terminus antibody. (B) The assay was repeated with the Erk inhibitor once it was identified as being the most potent inhibitor of myostatin expression to confirm results. (C) Western blot was performed on nuclear extracts to assess the levels of nuclear MEF-2. (D) EMSA was performed to assess MEF-2 DNA binding activity in nuclear extracts. #p<0.05 vs. PE (ANOVA); *p<0.05 vs. Con (ANOVA).

PE stress stimulates the hypertrophic response and activates growth and stress pathways in NCM's. This is accompanied by an increase in myostatin precursor protein and activated myostatin, which may exert negative feedback on the growth program. If the increase in myostatin is viewed as a response to stress, then it may be dependent on activation of stress and/or growth pathways. To test this hypothesis, NCM's were pretreated with small molecule inhibitors prior to PE stress to block activation of key growth and stress pathways in the heart, including PI3K-Akt, p38, Erk, and calcineurin-NFAT. Erk inhibition robustly blocked the upregulation of myostatin previously observed after 18 hours of PE stress (Fig. 4A), suggesting that Erk regulates myostatin expression following stress in NCM's. This finding was confirmed by repeating the assay with only the Erk inhibitor (Fig. 4B).

Since MAPK's have been shown to phosphorylate and activate the transcription factor MEF-2 [42] and since the myostatin promoter contains a MEF-2 responsive site [41], it was hypothesized that Erk regulates myostatin by phosphorylating MEF-2 and inducing nuclear translocation. Western blot of nuclear extracts from the inhibition assay demonstrate that increased MEF-2 is translocated to the nucleus following PE stress and that this effect is blocked by pretreatment with the Erk inhibitor (Fig. 4C). In addition, electrophoretic mobility shift assay (EMSA) results indicate that MEF-2 DNA binding activity is increased in nuclear extracts following PE stress, an effect that is also blocked by pretreatment with the Erk inhibitor (Fig. 4D).

### Effect of myostatin inhibition and overexpression on NCM size and number

**Figure 5:**
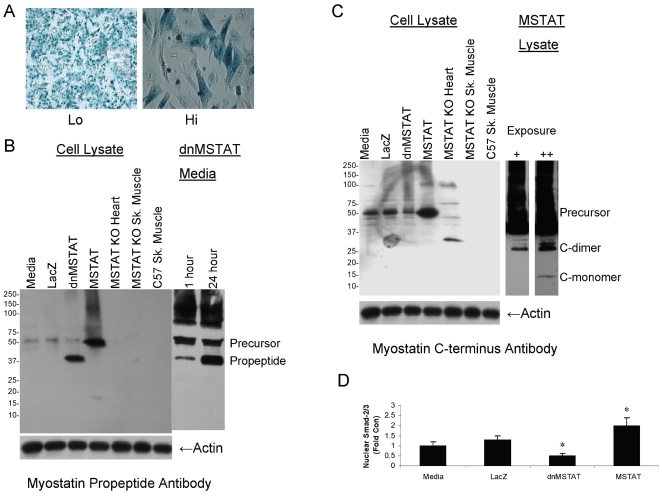
Myostatin manipulation in NCM culture. Cardiomyocytes were isolated from neonatal rats and grown to confluency. Cells were treated with Ad-CMV-LacZ (control), Ad-CMV-dnMSTAT, or Ad-CMV-MSTAT at MOI = 100 for 18 hours in the presence or absence of PE stress (100 uM). A media only control was also performed to control for viral effects. Each condition was tested n = 4 times. (A) X-gal histochemistry demonstrates high efficiency of adenovirus transfection. Scale bar represents 200 µm (lo) or 40 µm (hi). Western blot was performed to confirm expression of (B) dnMSTAT (propeptide antibody) and (C) MSTAT (C-terminus antibody). Samples from myostatin knock-out (MSTN KO) mouse heart and skeletal muscle, as well as from normal (C57) mouse skeletal muscle were also run as controls. Predicted molecular weights for the various forms of myostatin are as follows: full length precursor monomer (43–45 kDa); propeptide (30–32 kDa); C-terminus monomer (12–13 kDa); active C-dimer (24–26 kDa). Glycosylation may cause these forms to run approximately 5–10 kDa higher than predicted on PAGE. (D) Western blot was performed on nuclear extracts to quantify the levels of nuclear Smad2/3. *p<0.05 vs. Media and LacZ (ANOVA).

If myostatin is a negative regulator of NCM growth and proliferation, myostatin overexpression should decrease cell size and number, while myostatin inhibition should increase cell size and number. To test this hypothesis in vitro, NCM's were transfected with adenovirus expressing the following transgenes: LacZ (control), dominant negative myostatin (dnMSTAT), or myostatin (MSTAT) in the presence or absence of PE stress for 18 hours. A media only control was also performed to control for viral effects. Adenovirus mediated transfection of NCM's was very efficient. Figure 5A shows virtually 100% efficient cardiomyocyte transfection with the LacZ vector. Figure 5B demonstrates that the myostatin inhibitor (propeptide or dnMSTAT) is expressed in high levels in the NCM's and secreted into the media following transfection. Figure 5C confirms high level expression of myostatin precursor protein and biologically active C-dimer (and reduced C-monomer) compared to control following transfection.

Samples from myostatin knock out mouse (MSTN KO) heart and skeletal muscle, as well from normal mouse (C57) skeletal muscle, were run alongside the cell culture samples to determine the specificity of the antibodies. As demonstrated in Figure 5B, the propeptide antibody did not detect any bands in the mouse sample, despite equal protein loading (actin blot). This may reflect differential antibody affinity in rat cardiomyocytes vs. in vivo murine samples. However, bands of the expected sizes were detected in the cell culture samples: the 37 kDa propeptide band in the dnMSTAT sample and the 50 kDa myostatin precursor protein band in all lanes, with overexpression in the MSTAT sample.

Figure 5C shows that the C-terminus antibody detected the 50 kDA myostatin precursor protein band in all culture samples, with overexpression in the MSTAT sample. This band is absent in the MSTAT KO heart sample, confirming antibody specificity. With longer exposure, the C-dimer (∼25 kDa) and monomer (∼12.5 kDa) bands become apparent in the MSTAT sample. The C-terminus antibody did not detect bands in the skeletal muscle samples despite equal protein loading (actin blot), perhaps due to differential affinity of the antibody in heart vs. skeletal muscle samples.

Figure 5C also demonstrates that myostatin precursor protein is the predominant form of myostatin present compared to C-dimer. This phenomenon has been reported in cardiomyocyte culture previously [43]. Nuclear translocation (Fig. 5D) of Smad was decreased with myostatin inhibition and increased with myostatin overexpression.

**Figure 6:**
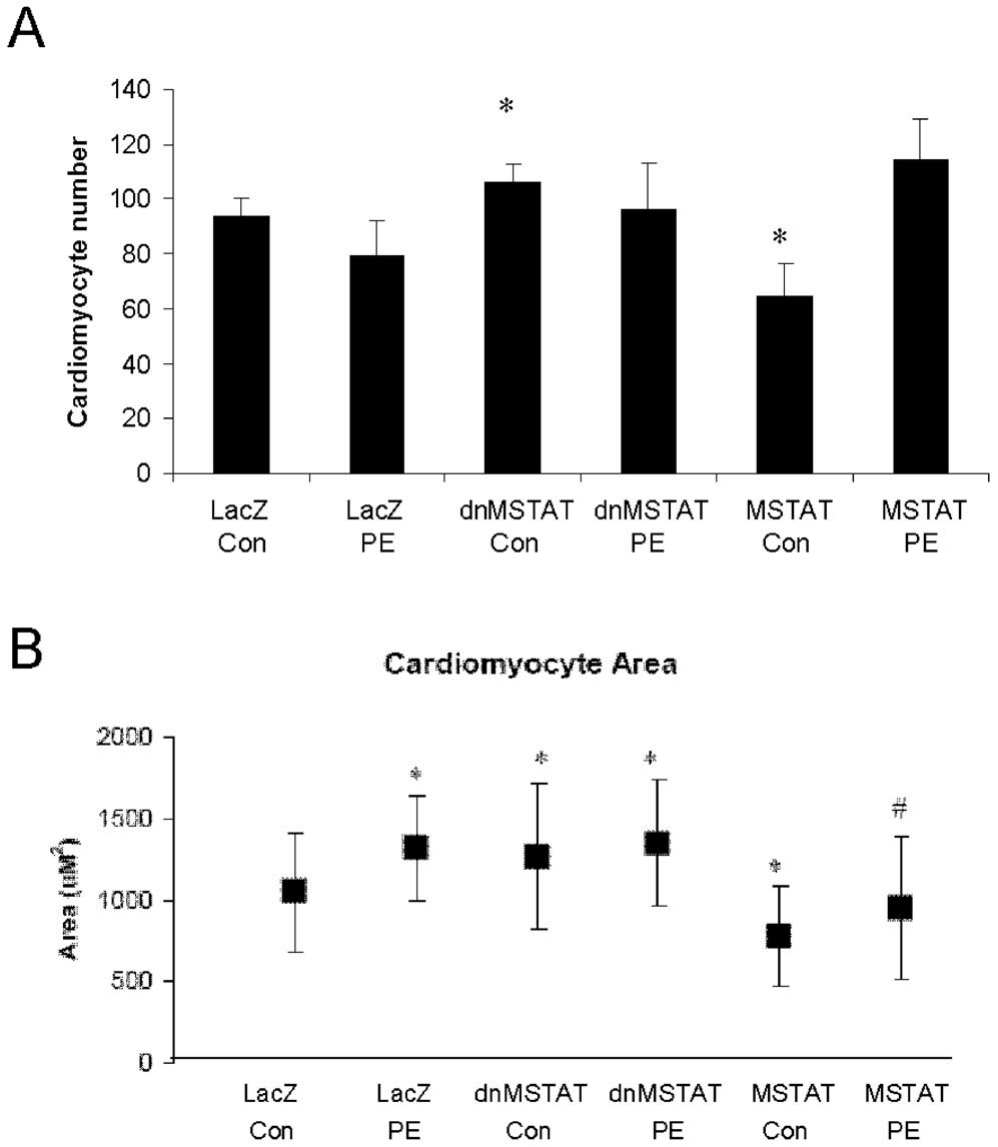
Myostatin negatively regulates NCM growth and proliferation. Cardiomyocytes were isolated from neonatal rats and grown to confluency. Cells were treated with Ad-CMV-LacZ (control), Ad-CMV-dnMSTAT, or Ad-CMV-MSTAT at MOI = 100 for 18 hours in the presence or absence of PE stress (100 uM). Each condition was tested n = 4 times. (A) Cell number was counted at 40X in 3 fields for each treatment. (B) NCM surface area was quantified in 3 fields at 40X using Fovea Pro software after laminin staining, and representative images are displayed. Approximately 500–800 cells were counted per condition. *p<0.05 vs. LacZ Con (ANOVA).

Figure 6A shows that cell number is increased by approximately 15% with myostatin inhibition and decreased by approximately 20% with myostatin overexpression. PE had no effect on cell proliferation in the control and myostatin inhibition groups but countered the decrease in proliferation observed with myostatin overexpression. Figure 6B demonstrates that myostatin inhibition induced an approximately 20% increase in cell size, while myostatin overexpression resulted in an approximately 20% decrease in cell size. PE stress induced an increase in cell size in controls with a magnitude similar to that observed with myostatin inhibition, but was not additive to the effect of myostatin inhibition. However, myostatin overexpression blocked PE-induced cell growth. These data indicate that myostatin negatively regulates NCM proliferation and size.

### Effect of myostatin inhibition and overexpression on signal transduction through growth pathways in NCM's

**Figure 7:**
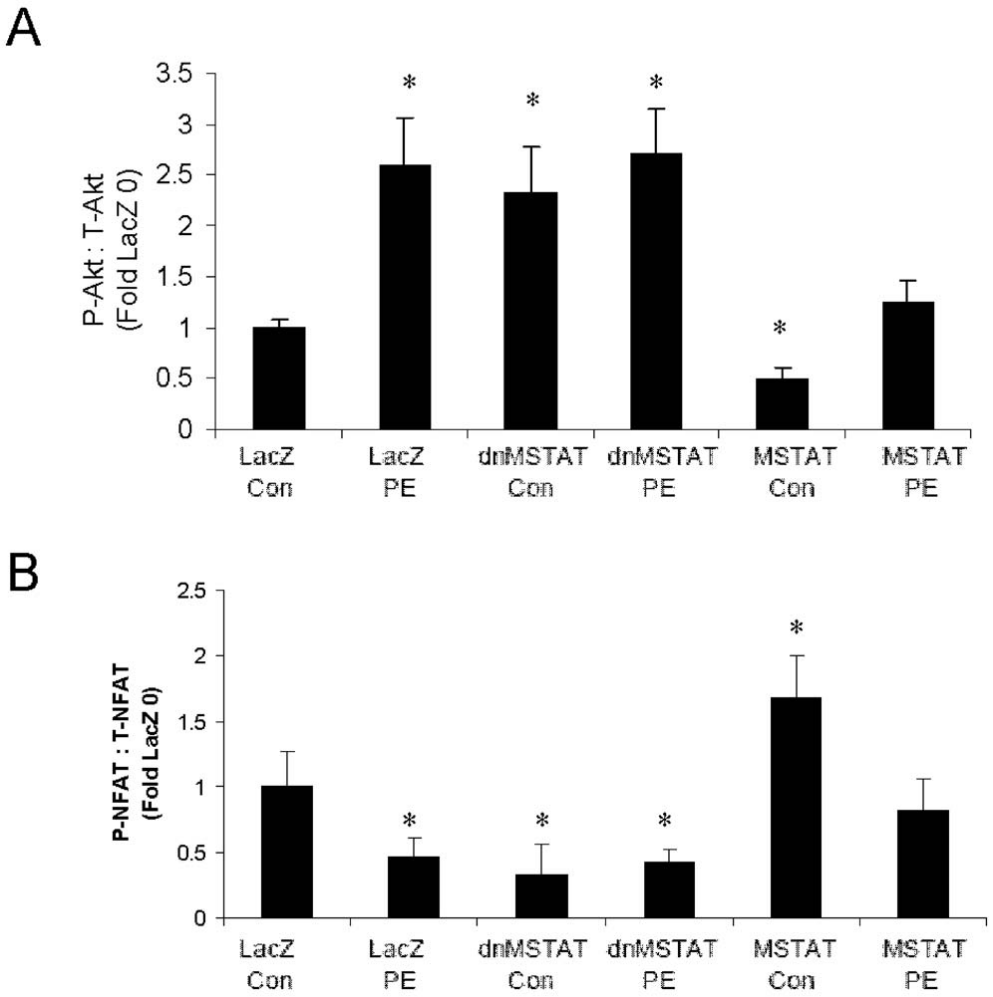
Myostatin negatively regulates NCM growth and proliferation by modulating signaling through Akt and NFAT3. NCM's were cultured as described above. Cells were treated with Ad-CMV-LacZ (control), Ad-CMV-dnMSTAT, Ad-CMV-MSTAT at MOI = 100 for 18 hours, then stressed with PE (100 uM) for 0 or 15 minutes. Each condition was tested n = 4 times. Phospho-specific Western blot was performed to assess the activation states of (A) Akt and (B) NFAT3. *p<0.05 vs. LacZ Con (ANOVA).

Myostatin may negatively regulate NCM growth by blocking signaling through cardiac growth pathways. To test this hypothesis in vitro, NCM's were transfected with adenovirus expressing LacZ, dnMSTAT, and MSTAT as previously described, then stressed with PE or media control for 15 minutes. Phosphorylation states of key signaling proteins (Akt, NFAT3) were evaluated. Myostatin inhibition caused increased activation of Akt and NFAT3 (activated by dephosphorylation) compared to LacZ control, while myostatin overexpression caused decreased activation of Akt and NFAT3 compared to LacZ control (Fig. 7A, B). PE stress induced activation of Akt and NFAT3 in LacZ-treated NCM's to levels similar to those observed with myostatin inhibition but had no additive effect in dnMSTAT-treated cells (Fig. 7A, B). Myostatin overexpression blocked PE-induced activation of Akt (phosphorylation) and NFAT3 (dephosphorylation) (Fig. 7A, B). These results support the hypothesis that myostatin regulates NCM growth by blocking activation of growth pathways.

### Effect of myostatin inhibition and overexpression on cardiac growth in vivo

It is hypothesized that myostatin negatively regulates cardiac growth. To test this hypothesis in vivo, neonatal C57 mice were injected with adeno-associated virus (AAV9) expressing the following transgenes: LacZ (control), dominant negative myostatin (dnMSTAT), or myostatin (MSTAT), and followed for 6 and 12 weeks. A saline injection group was used to control for viral effects.

**Figure 8:**
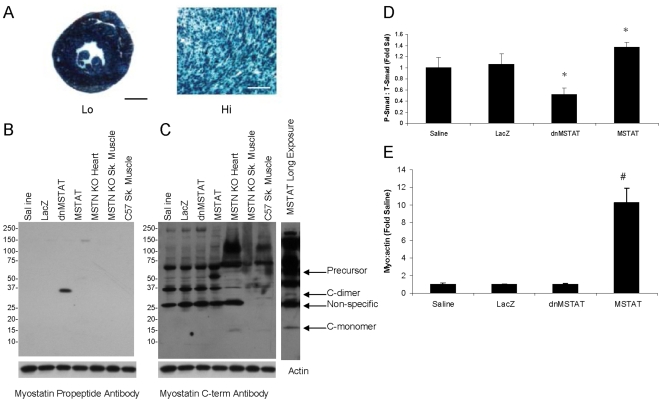
Myostatin manipulation in vivo. Neonatal C57 mice (day 4–5) were treated with either Saline (n = 12), AAV9-LacZ (n = 11), AAV9-dnMSTAT (n = 12), or AAV9-MSTAT (n = 8),via subxiphoid injection to target the heart. A saline injection group was added to control for viral effects. (A) X-gal histochemistry demonstrates distribution of transgene expression. Western blot demonstrates expression of (B) dominant negative myostatin (propeptide antibody), (C) myostatin precursor (∼50 kDa) and processed C-monomer (∼13 kDa) (C-terminus antibody), and (D) phospho-Smad2/3. Samples from myostatin knock-out (MSTN KO) mouse heart and skeletal muscle, as well as from normal (C57) mouse skeletal muscle were also run as controls. The precursor protein is depicted with an arrow at 50 kDa and is present at high levels in the MSTN lane and absent in the MSTN KO lanes. Proposed C-dimer and monomer bands are also depicted with an arrow. Predicted molecular weights for the various forms of myostatin are as follows: full length precursor monomer (43–45 kDa); propeptide (30–32 kDa); C-terminus monomer (12–13 kDa); active C-dimer (24–26 kDa). Glycosylation may causes these forms to run approximately 5–10 kDa higher than predicted on PAGE. (E) Quantification of myostatin full-length precursor (∼50 kDa). *p<0.05 vs. Saline and LacZ (ANOVA). #p<0.05 vs. Saline, LacZ, and dnMSTAT (ANOVA). Scale bar represents 1 mm (lo) or 200 µm (hi).

Efficiency of transgene expression was very high. B-galactosidase histochemistry demonstrates that the transgene is distributed throughout the heart in nearly 100% of the cardiomyocytes (Fig. 8A). Western blot demonstrates expression of dominant negative myostatin (37 kDa) (Fig. 8B) and myostatin (precursor at ∼50 kDa and C-dimer and monomer at ∼25 kDa and ∼13 kDa, respectively) (Fig. 8C) and confirms decreased and increased Smad phosphorylation with myostatin inhibition and overexpression, respectively (Fig. 8D). Myostatin precursor was overexpressed at approximately 10 fold the baseline level of expression detected in the Saline, LacZ, and dnMSTAT groups (Fig. 8E).

Samples from myostatin knock out mouse (MSTN KO) heart and skeletal muscle, as well from normal mouse (C57) skeletal muscle, were run alongside the experimental samples to determine the specificity of the antibodies. The propeptide antibody (Fig. 8B) only detected the 37 kDa propeptide band that was overexpressed in the dnMSTAT sample. No bands (myostatin precursor protein or otherwise) were apparent in the other lanes, suggesting that this antibody has relatively low affinity for endogenous murine myostatin. The actin blot demonstrates equal protein loading.

The C-terminus antibody (Fig. 8C) was relatively non-specific in the murine samples. Multiple bands are present in both experimental and MSTN KO lanes, suggesting that these bands are non-specific. However, the 50 kDa myostatin precursor protein band was present at low levels in the Saline, LacZ, and dnMSTAT lanes with 10 fold overexpression (Fig. 8E) in the MSTAT lane. This band was absent in the MSTAT KO lanes, confirming its specificity as the myostatin precursor protein. With longer exposure, bands at ∼25 kDa (the lighter band just above the strong non-specific band detected in all cardiac sample, including the MSTN KO sample) and ∼13 kDa become apparent in the MSTAT sample (Fig. 8C), which correspond to myostatin C-dimer and monomer, respectively. Bands of corresponding size are found in the normal (C57) mouse skeletal muscle sample but not the MSTN KO samples, suggesting that they are specific myostatin bands. It should also be noted that a diffuse, artifactual band appears in the MSTN KO heart lane at a level just above the ∼13 kDa C-monomer band in the MSTN and C57 skeletal muscle lanes. The diffuse appearance of this band and the fact that it runs slightly higher than the proposed C-monomer bands in the MSTN and C57 skeletal muscle lanes suggests that is it not a myostatin related band. Figure 8C also demonstrates that the major form of myostatin present in the heart is the precursor protein (50 kDa), while the major form of myostatin in skeletal muscle is activated myostatin (C-dimer and monomer), with very little precursor protein present.

**Figure 9:**
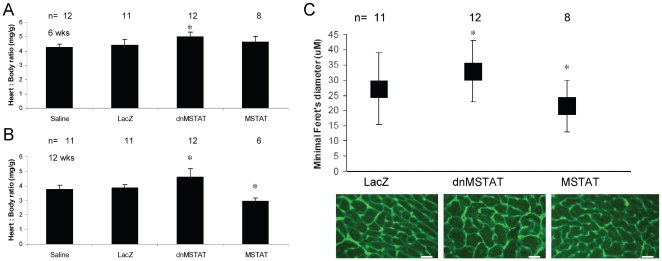
Myostatin negatively regulates cardiac size in vivo. Neonatal C57 mice (day 4–5) were treated with either Saline, AAV9-LacZ, AAV9-dnMSTAT, or AAV9-MSTAT via subxiphoid injection to target the heart. Mice were followed for 6 and 12 weeks, and the n for each condition is reported on the figure. Relative heart weight is reported as the ratio of heart weight to body weight at (A) 6 weeks and (B) 12 weeks. (C) Cardiomyocyte size at 12 weeks is reported as minimal feret's diameter and was measured in 3 fields using Fovea Pro software 3 months following treatment. Approximately 500–800 cells were counted per condition. *p<0.05 vs. Saline and LacZ (ANOVA). Representative images are displayed. Scale bar represents 40 µm. Magnification is 40X.

At 6 weeks, myostatin inhibition induced an approximately 15% increase in relative cardiac mass (heart to body) compared to control (Fig. 9A). Myostatin overexpression had no effect at 6 weeks (Fig. 9A). At 12 weeks, myostatin inhibition induced an approximately 20% increase in relative cardiac mass compared to control, and myostatin overexpression induced an approximately 20% decrease in relative cardiac mass (Fig. 9B). These data support a role for myostatin in the control of cardiac mass in vivo.

Cardiomyocyte size was increased by approximately 20% in the myostatin inhibition group, while size was decreased by approximately 20% in the myostatin overexpression group at 12 weeks (Fig. 9C). This suggests that alterations in cardiac mass were primarily due to changes in cell size and not number in our model.

### Effect of myostatin inhibition and overexpression on cardiac function

Neither myostatin inhibition nor myostatin overexpression had any effect on cardiac function of C57 mice as measured by 2-D echocardiography (Table 1).

**Table 1:** Cardiac function as assessed by 2-D echocardiography at 3 months post treatment.

	n	HR(bpm)	IVSd(cm)	LVIDd(cm)	LVFW(cm)	LVIDs(cm)	FS(%)	EDV(ml)	ESV(ml)	EF(%)	CO(L/min)	SV(ml)
Saline	10	275±71	0.08±0.01	0.36±0.03	0.09±0.01	0.21±0.03	39±6.7	0.12±0.03	0.03±0.01	75±7.1	0.02±0.01	0.09±0.02
LacZ	11	323±74	0.08±0.01	0.30±0.04	0.08±0.01	0.18±0.04	42±9.6	0.08±0.03	0.02±0.01	78±10	0.02±0.01	0.06±0.02
dnMSTAT	8	262±39	0.08±0.01	0.34±0.06	0.09±0.01	0.20±0.05	40±10	0.11±0.05	0.03±0.02	76±12	0.02±0.01	0.08±0.04
MSTAT	6	311±99	0.08±0.01	0.35±0.03	0.09±0.02	0.21±0.04	40±12	0.11±0.03	0.03±0.01	76±13	0.03±0.01	0.09±0.03

p = NS for all parameters.

HR, heart rate; bpm, beats per minute; IVSd, interventricular septum in diastole; LVIDd, left ventricular inner diameter in diastole; LVFW, left ventricular free wall; LVIDs, left ventricular inner diameter in systole; FS, fractional shortening; EDV, end diastolic volume; ESV, end systolic volume; EF, ejection fraction; CO, cardiac output; SV, stroke volume.

### Effect of myostatin inhibition and overexpression on Akt and NFAT3 activity

**Figure 10:**
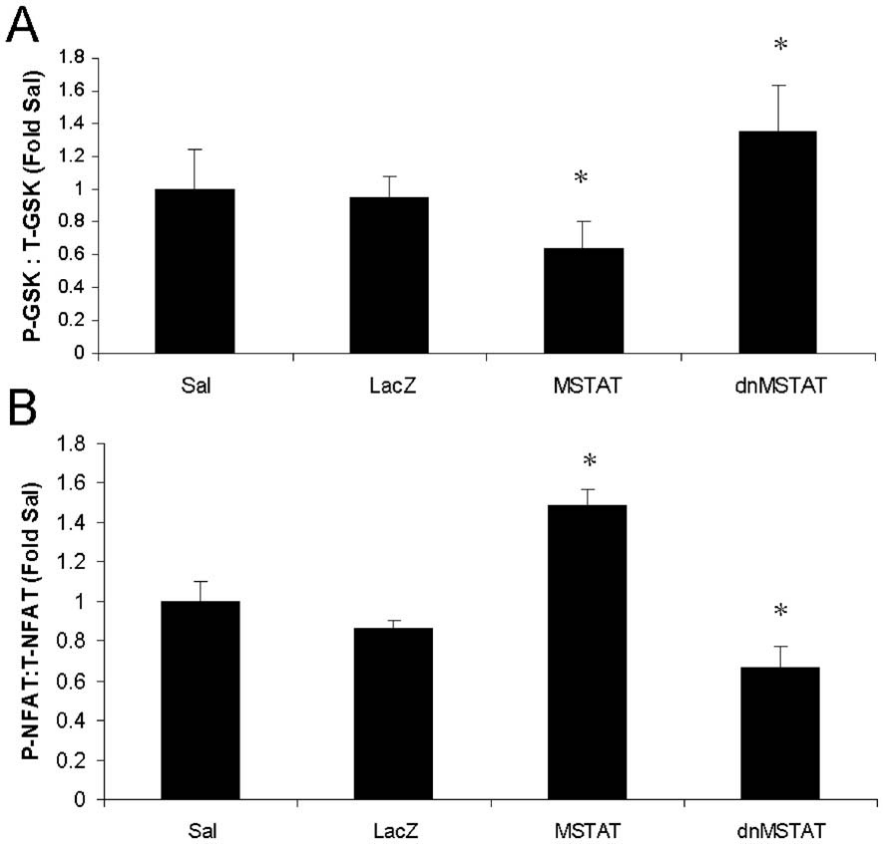
Myostatin modulates signaling through Akt and NFAT3 pathways in vivo. Neonatal C57 mice (day 4–5) were treated with either Saline (n = 12), AAV9-LacZ (n = 11), AAV9-dnMSTAT (n = 11), or AAV9-MSTAT (n = 8) via subxiphoid injection to target the heart. Phospho-specific Western blot was performed to determine (A) Akt activity (via GSK-3β phosphorylation) and (B) NFAT3 activity. *p<0.05 vs. Saline and LacZ (ANOVA).

Myostatin may negatively regulate NCM growth by blocking signaling through cardiac growth pathways. Western blot performed on cardiac tissue lysates demonstrates that Akt and NFAT3 activity (decreased by phosphorylation) are increased in mice in the myostatin inhibition group and decreased in mice in the myostatin overexpression group (Fig. 10A, B). This data confirms our findings in NCM culture that myostatin can regulated cardiac growth by modulating signaling through Akt and NFAT3.

## Discussion

**Figure 11:**
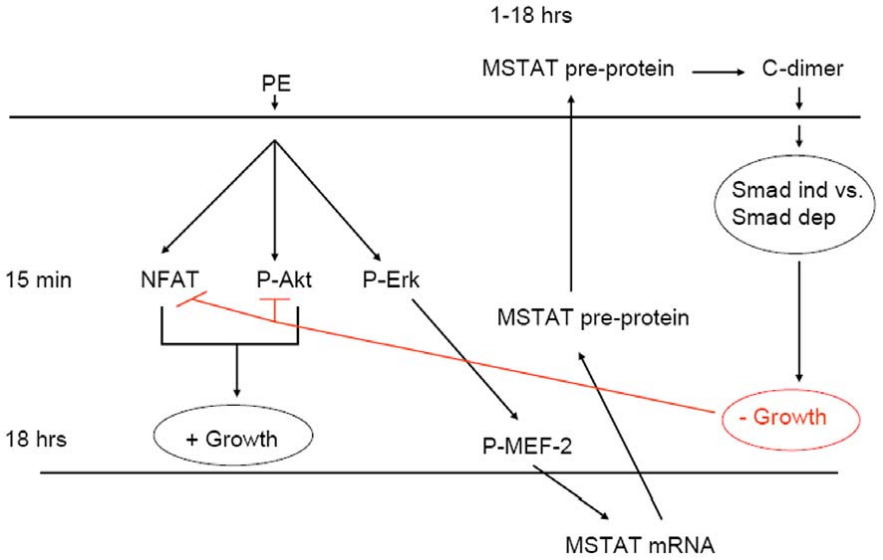
Model of myostatin regulation and function in the heart. In this study, PE was used to model cardiac stress. This led to early (15 minute) activation of the cardiac growth pathways NFAT and Akt, and an increase in cell size was apparent by 18 hours. Erk was also activated early (15 minutes), and this was shown to be necessary for subsequent MEF-2 phosphorylation and increase in myostatin expression. Myostatin was shown to be able to exert negative feedback on growth by blocking activation of NFAT and Akt. In this model, therefore, PE activates growth via NFAT and Akt, while at the same time activating Erk, which drives MEF-2 dependent upregulation of myostatin, which in turns exerts negative feedback on growth by blocking NFAT and Akt activation. Further studies are needed to determine the mechanism of myostatin-mediated blockade of Akt and NFAT activation, including whether each process is Smad independent vs. Smad dependent.

Myostatin is a negative regulator of skeletal muscle mass, but its role in cardiac physiology is much less clear. Since it is expressed in the heart, it has been suggested that myostatin may perform a similar function there [25]. In this study, we report data to support the hypothesis that myostatin is upregulated in response to cardiac stress as part of a negative feedback loop to control the hypertrophic response. This response appears to be Erk dependent. In addition, our data support the hypothesis that myostatin negatively regulates cardiac growth by modulating signaling through the cardiac growth pathways Akt and NFAT. A detailed model is depicted in Figure 11.

In the first set of experiments, we established that PE is an effective cardiac stressor in vitro. Treatment of NCM's with PE resulted in activation of growth (Akt/GSK3-β, NFAT) and stress (Erk) pathways. This was accompanied by an increase in cell size but not cell number. Similar results have been observed following PE stress of NCM's [34].

We next examined myostatin expression and activation following PE stress in NCM culture. PE stress induced activation of myostatin by 60 minutes, and by 18 hours, levels of myostatin precursor protein were also increased. Since myostatin is a TGF-β family member known to signal through the Smad pathway [4], we confirmed myostatin activation by documenting Smad phosphorylation. However, it should be noted that upregulation of other TGF-β family members during cardiac stress may also contribute to the observed increase in Smad phosphorylation, as it has been previously reported that ligands other than myostatin can signal through the “myostatin” receptor (ActIIBR) [7]. Myostatin upregulation has also been observed following cyclic stretch of NCM's [27] and treatment of NCM's with angiotensin-II [42], [43]. Myostatin may be upregulated to exert negative feedback on the growth response initiated by these stressors [44].

If this is true, the myostatin upregulation observed following PE stress may be dependent on upstream activation of growth and/or stress pathways. Our data demonstrate that blocking Erk activation with a small molecule inhibitor prior to PE stress blocks the previously observed increase in myostatin expression. Therefore, it appears that myostatin upregulation following PE stress is Erk dependent. In this context, myostatin may be viewed as part of the cell's response to stress. To our knowledge, this is the first report of Erk-mediated regulation of myostatin in the heart. In addition, since previous studies have reported that myostatin regulation following either IGF-1 or angiotensin-II stress of NCM's is p38 dependent [27], [43], myostatin regulation following cardiac stress may be stressor-specific.

Since myostatin has a MEF-2 responsive element in its promoter [41] and since MAPK's are known to phosphorylate and activate MEF-2 [42], Erk may regulate myostatin via MEF-2 activation. We observed that both nuclear translocation and DNA binding activity of MEF-2 are increased following PE stress and that these effects are blocked by pretreatment of NCM's with an Erk inhibitor prior to PE stress. This supports our hypothesis that Erk mediates an increase in myostatin expression via increased MEF-2 activity. MEF-2 has been implicated in the regulation of myostatin in NCM's following other means of stress, including stretch [27] and AT-II [43], and in vivo following induction of an aorta-caval shunt [27]. However, since the degree of inhibition of MEF-2 DNA binding activity following Erk inhibition is relatively small compared to the inhibition of myostatin expression, other transcription factors likely contribute to the regulation of myostatin expression in addition to MEF-2. Further investigation is needed to identify these factors.

In the next set of experiments, we tested the hypothesis that myostatin negatively regulates cardiomyocyte size by modulating signaling through growth pathways. NCM's were treated with adenovirus expressing dnMSTAT, MSTAT, or LacZ and then stressed with PE or media only. The dominant negative construct that was used to inhibit myostatin is a modified form of Myostatin Belgian Blue, named after the bovine strain harboring this mutation. This construct acts as a dominant negative because the Belgian Blue mutant contains an 11 bp deletion that causes a premature stop in the C-terminus, leaving only the inhibitory propeptide to be expressed [22]. In addition, it contains a D76A mutation, which makes the inhibitory propeptide resistant to proteolysis [3]. In effect, this construct is a protease-resistant propeptide that can bind to and inhibit endogenous myostatin by preventing receptor binding [1].

We observed that myostatin inhibition induced an approximately 20% increase in cardiomyocyte size, similar to what has been reported by another investigator [34]. PE stress induced a similar increase in size in LacZ treated cells, but did not augment growth in dnMSTAT treated cells. These data suggest that myostatin and PE may exert effects on cardiomyocyte size by modulating signaling through the same pathways, albeit in opposite directions. For example, myostatin may inhibit a specific pathway, while PE may activate it. The absence of an additive increase in cell size with the combination of PE stress and myostatin inhibition may indicate that our potent, protease-resistant myostatin inhibitor and PE stress independently achieved maximal pathway activation at the doses administered. In that case, no further activation or increase in size would be possible when both treatments were combined. Conversely, myostatin overexpression induced an approximately 20% decrease in cardiomyocyte size and blocked the hypertrophic response to PE. Taken together, these data strongly support the hypothesis that myostatin is a negative regulator of cardiomyocyte size.

We also observed that myostatin inhibition promoted NCM proliferation and that myostatin overexpression blocked NCM proliferation. Data from other investigators suggests that myostatin regulates cardiomyocyte proliferation by targeting the cell cycle proteins p21 and cdk2 [28], [32]. Further investigation is needed in our model to confirm these results.

In the next line of experiments, we sought to identify the pathways with which myostatin may cross-talk to regulate cardiomyocyte size. Since our data suggested that myostatin may interact with the same pathways as PE, we investigated two pathways that were activated by PE in our model: Akt and NFAT3. We found that myostatin inhibition resulted in increased activation of Akt and NFAT3, while myostatin overexpression caused decreased activation of Akt and NFAT3. Similar to was observed with cardiomyocyte size, PE stress did not induce additional activation of Akt and NFAT3 in combination with myostatin inhibition, but myostatin overexpression did block PE-induced activation of these pathways. These data suggest that myostatin may negatively regulate cell size by blocking activation of Akt and NFAT3 in cardiomyocytes. It has been previously reported that myostatin can modulate Akt signaling in NCM's [34], perhaps by upregulating or stabilizing PTEN [37]. Although another group has suggested that myostatin may cross-talk with NFAT2 in skeletal muscle [38], [39], this is the first report of cross talk between myostatin and NFAT3 pathways in cardiomyocytes. It is possible that myostatin signaling activates kinases capable of phosphorylating NFAT3, thus countering the effects of calcineurin, or it may be that mediators of myostatin signaling further downstream can suppress calcinuerin at the transcriptional level. Further investigation is needed to identify the link between myostatin and NFAT3.

In the next set of experiments, we tested the hypothesis that myostatin negatively regulates cardiac growth in vivo. AAV9 was used to express LacZ, dnMSTAT, or MSTAT in mice, and a saline group was added to control for viral effects. Mice were followed for 6 and 12 weeks. AAV9 was used because it is superior in the rodent heart compared to other serotypes [45], [46], [47], [48]. Myostatin inhibition in the heart resulted in increased relative cardiac mass at 6 and 12 weeks, while myostatin overexpression resulted in decreased relative cardiac mass at 12 weeks only. Decreased mass may not have been induced with myostatin overexpression until 12 weeks because the myostatin protein undergoes extensive post-translational modification before full activity is achieved, and once active, is inhibited by several local and circulating factors, including follistatin, FLRG, GASP-1, and endogenous myostatin propeptide [1]. This is in contrast to the dnMSTAT construct used in this study, which simply required translation and secretion.

These alterations in cardiac mass were accompanied by a corresponding increase or decrease in cardiomyocyte size and Akt and NFAT3 activity in the myostatin inhibition and overexpression groups, respectively. These results suggest that myostatin plays an important role in negatively regulating cardiac growth in vivo and that it appears to do so by modulating signaling through key cardiac growth pathways. In addition, since the alterations in individual cardiomyocyte size were proportionally similar to the overall alteration in total cardiac mass, it suggests that myostatin exerted its effects primarily via hypertrophy and not via hyperplasia in these post-natal hearts.

These data are in agreement with a recent study that reports similar changes in cardiac size in myostatin transgenic and knock out mice [32]. However, a controversy exists over the size of myostatin null hearts with some investigators reporting decreased size [33], [34], others reporting increased size [32], and still others reporting no effect on size [35]. This discrepancy may exist due to the fact that the genetic background of the mice is not consistent from study to study. It is obvious from recent studies that at least one other unidentified factor has a significant influence on the regulation of skeletal muscle mass since the myostatin null mouse will experience a further increase in muscle mass if treated with soluble activin IIB receptor [7] or crossed with a follistatin transgenic line [49]. This unknown factor may compensate or over-compensate for the absence of myostatin to various degrees in different mouse strains, and this may ultimately affect cardiac size.

It should also be noted that the mice in our study are different from the myostatin null mice described by other investigators. Myostatin null mice never express myostatin and may experience over-compensation of cardiac growth regulation during embryonic and postnatal development that results in a smaller heart. Indeed, lack of myostatin during the embryonic period may have a permanent effect on cardiac size and/or regulation of cardiac size since it was recently demonstrated that myostatin is dynamically regulated during embryonic development [28]. Therefore, the effect of myostatin knock-out may be very different from the effect of myostatin inhibition postnatally, which is the treatment regimen being used in the clinical trials of myostatin inhibition [36]. In contrast, the mice in this study were treated in the postnatal period. They experienced normal embryonic development, and their response to myostatin inhibition during the postnatal period was an increase in cardiac mass, supporting a role for myostatin in negative regulation of cardiac growth in the postnatal period.

We next examined the effect of myostatin manipulation on cardiac function by performing 2-D echocardiography at 3 months and observed no alteration of cardiac function. This finding is in agreement with several other studies [32], [34], [35]. It has been suggested that just as in skeletal muscle, myostatin may increase cardiac size without affecting the functional efficacy of the contractile units [32]. Further studies are needed to evaluate the effects of myostatin manipulation in the failing heart.

In summary, the results of this study suggest that myostatin is an important negative regulator of cardiac growth. Myostatin is upregulated in an Erk-dependent manner following cardiomyocyte stress, and it regulates cardiac growth by modulating signaling through important growth pathways in the heart, including Akt and NFAT3. These findings have potential clinical implications that mandate further investigation. Clinical trials using myostatin inhibition via an antibody approach as a potential treatment for several types of muscular dystrophy are ongoing [36]. Since many of these patients can be expected to experience some form of cardiomyopathy in the future, it will be important to discern how myostatin inhibition will impact the injured heart. If myostatin plays a critical role in regulating cardiac remodeling following stress, inhibition of myostatin in this population, or in any population experiencing cardiac stress, could result in a very deleterious outcome. Large animal studies in which myostatin is both inhibited and overexpressed in the setting of cardiac failure should be the next step to address these important issues.

## Materials and Methods

### Ethics Statement

All animals were handled in compliance with the *Guide for the Care and Use of Laboratory Animals* published by the National Institutes of Health (NIH publication No. 85–23, revised 1996). In addition, all animal work was performed under protocols approved by the Institutional Animal Care and Use Committee of the University of Pennsylvania.

### Vector Production

Each adenovirus vector was designed to express either full-length murine myostatin (MSTAT), dominant negative murine myostatin (dnMSTAT), or the LacZ reporter gene under control of the constitutive cytomegalovirus (CMV) promoter. Vectors were produced according to the previously described protocol by the Vector Core of the University of Pennsylvania [50].

Each adeno-associated virus serotype 9 (AAV9) vector was designed to express either full-length murine myostatin (MSTAT), dominant negative murine myostatin (dnMSTAT), or the nuclear-localized LacZ reporter gene under control of the constitutive truncated desmin promoter with synthetic enhancer (SEDES460 promoter). Vectors were produced according to the previously described pseudotyping protocol by the Vector Core of the University of Pennsylvania [51].

**Figure 12:**
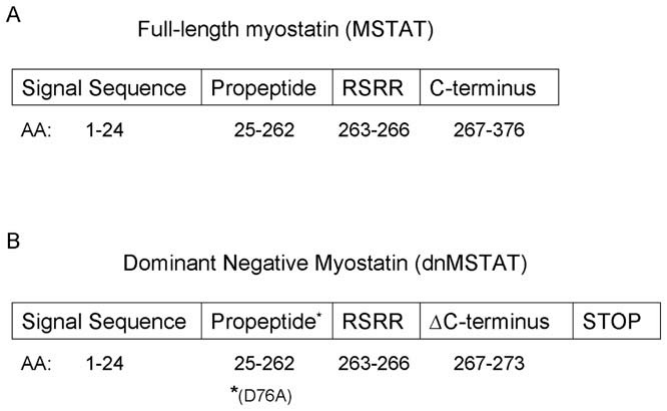
Schematic of domains included in each myostatin construct. (A) The cDNA encoding full-length murine myostatin (MSTAT) that was overexpressed from both adenovirus and AAV vectors contained the following domains: signal sequence, propeptide (N-terminus) containing aspartate at amino acid 76 (D76), RSRR cleavage site, and C-terminus. Myostatin activation requires cleavage at RSRR as an initial step to disrupt the covalent bond between the C-terminus and the propeptide. However, the inhibitory propeptide continues to associate with the C-terminus non-covalently, and full activation of myostatin requires a second cleavage at D76 to free the C-terminus from the propeptide and induce propeptide degradation. (B) The cDNA encoding dominant negative murine myostatin (dnMSTAT) that was overexpressed from both adenovirus and AAV vectors contained the following domains: signal sequence, propeptide (N-terminus) containing a D76A mutation, RSRR cleavage site, and a truncated C-terminus resulting from a premature stop. As a result, no full-length or active myostatin is expressed from this construct. In contrast, a protease-resistant (D76A) inhibitory propeptide that acts in a dominant negative manner is expressed. Corresponding amino acid (AA) positions are indicated below each cartoon. Note that cartoon is not to scale.

The MSTAT transgene contains the cDNA for the entire 1131 bp murine myostatin gene encoding 376 amino acids (aa). This includes a 24 aa signal sequence, a 242 aa inhibitory N-terminal propeptide, and a 110 aa C-terminus, the dimer of which is biologically active myostatin (Fig. 12A). The dnMSTAT transgene contains the cDNA for the first 822 bp (274 aa) of the murine myostatin gene followed by a premature stop. This is modeled after the mutation in the double-muscled Belgian Blue cow [52]. It also contains an A to C transition at bp 227 (D76A), which destroys a cleavage site and confers protease resistance (Fig. 12B) [3]. In effect, the dnMSTAT construct is a protease-resistant inhibitory propeptide lacking the biologically active C-terminus that is designed to inhibit endogenous myostatin.

### Primary Cardiomyocyte Culture

Primary cultures of rat neonatal cardiomyocytes (NCM's) were prepared from Wistar rats as previously described [53]. 2×10^6^ cells were plated per 60 mm dish. The cardiac stress response was induced using phenylephrine (PE) at 100 uM (Sigma, St. Louis, MO). To determine the roles of Erk, p38, PI3K, and calcineurin in PE-induced myostatin expression, NCM's were pretreated for 1 hour before PE stress with the following inhibitors: 50 uM PD98059 (Erk, Calbiochem, San Diego, CA); 3 uM SB203580 (p38, Calbiochem, San Diego, CA); 5 nM wortmannin (PI3K, Sigma, St. Louis, MO); or 1 uM cyclosporine (calcineurin, Sigma, St. Louis, MO). For overexpression studies, adenovirus constructs were used at an MOI = 100.

### Mouse Protocol

Neonatal mice were injected with vector or saline control as previously described [48], [54], [55]. Briefly, four to five day old C57/Bl6 mice underwent cryoanesthesia, and a puncture was made at the left costoxiphoid angle of the anterior chest with a 33 gauge Hamilton needle. 50 uL containing 2.5×10^11^ genome copies of the AAV9 vector in normal saline were then injected into the pericardial space via this subxiphoid approach. Pups were subsequently rewarmed under a heat lamp and returned to their mothers for further care. At weaning, males and females were separated, and only males continued in the study. The final n per group ranged from 5–12 and is reported in the appropriate figure or table legend. Euthanasia was scheduled for 6 weeks and 12 weeks.

### Western Blot

Cardiac samples obtained for Western blotting were snap-frozen in liquid nitrogen after removal from the mouse without OCT fixative. Specimens were pulverized, homogenized in 10 volumes of triple-detergent lysis buffer [50 mM Tris, pH 8.0, 0.1% SDS, 1.0% Triton X-100, 0.5% DOC, 5 mM EDTA, 50 mM DTT, 0.4 tab/10 mL Complete protease inhibitor (Roche, Indianapolis, IN)], and centrifuged at 13,000 rpm for 5 minutes. NCM's were harvested in 150 uL of lysis buffer and centrifuged as above. Protein concentration of the supernatant was then determined using the BioRad Protein Assay (Hercules, CA). Either 50 µg (whole cell lysate from NCM culture or intact hearts) or 15 ug [nuclear extract from NCM culture, prepared using the Pierce (Rockford, IL) NE-PER kit] of each sample of serum were electrophoresed on a 4–20% SDS-polyacrylamide (Lonza, Rockland, ME) gel following the addition of 2X sample loading buffer (130 mM Tris, pH = 8.0, 20% glycerol, 4.6% SDS, 2% DTT, 0.02% bromophenol blue) and 5 minutes of denaturation at 100°C. Proteins were then transferred to Immobilon-P (Millipore, Bedford, MA) using the iBlot transfer apparatus (Invitrogen, Carlsbad, CA). The membrane was subsequently blocked with 5% nonfat dry milk in Tris-buffered saline containing 0.05% Tween 20. Immunoblotting was performed to detect C-term myostatin (polyclonal) (1∶500, Millipore, AB3239, Temecula, CA); N-term myostatin (polyclonal) (R&D Systems, AF1539, Minneapolis, MN); phospho-Smad 2/3 (polyclonal) (1∶500, Millipore, Temecula, CA); Smad 2/3 (polyclonal) (1∶1000, Cell Signaling Technology, Danvers, MA); MEF-2 (polyclonal) (1∶1000, Santa Cruz Biotechnology, Santa Cruz, CA); phospho-Erk1/2 (polyclonal) and total Erk1/2 (polyclonal) (1∶1000, Cell Signaling Technology, Danvers, MA); phospho-GSK-3β (monoclonal) and total GSK-3β (polyclonal) (1∶1000, Cell Signaling Technology, Danvers, MA); phospho-Akt (polyclonal) and total Akt (polyclonal) (1∶500, 1∶1000, Cell Signaling Technology, Danvers, MA); NFAT3 (polyclonal) (1∶500, Santa Cruz Biotechnology, Santa Cruz, CA); and actin (monoclonal) (1∶2000, Sigma, St. Louis, MO). Detection was performed using the Super Signal West Pico Chemiluminescent Substrate kit (Pierce, Rockford, IL). Protein bands were quantified using densitometry software (Alpha Innotech, San Leandro, CA), and bands were normalized to the actin band as a loading control. Phospho-protein bands were normalized to the relevant total protein (i.e., phospho-Smad to total Smad).

### Electrophoretic Mobility Shift Assay (EMSA)

Nuclear extracts were prepared from NCM's using the NE-PER kit (Pierce, Rockford, IL). Consensus and mutant control oligonucleotides for Smad-2 [56] and MEF-2 [43] were prepared by Integrated DNA Technologies (Coralville, IA); biotin labeled using the Biotin 3′ End DNA Labeling Kit (Pierce, Rockford, IL), and annealed into doubled-stranded probes for 1 hour at room temperature. The EMSA was perfomed using the Lightshift Chemiluminescent EMSA kit (Pierce, Rockford, IL). Briefly, 2 ug nuclear extract were used per reaction, and reactions were electrophoresed on a 6% DNA Retardation Gel (Invitrogen, Carlsbad, CA) and transferred to a Biodyne B nylon membrane (Pierce, Rockford, IL). Controls were performed with no extract, excess unlabelled consensus probe, and excess labeled mutant probe to assess the specificity of the assay. Specificity was also confirmed by observing antibody-induced shift of the DNA/protein complex.

### Histology and Cardiomyocyte Size Analysis

NCM's were fixed with 2% paraformaldehyde/0.1% glutaraldehyde for 5 minutes, washed in PBS, and blocked with 5% BSA in PBS for 15 minutes. Slides were incubated with an anti-laminin antibody (1∶100, Neomarkers, Fremont, CA) for 1 hour at room temperature. Slides were washed with PBS; incubated with anti-rabbit-488 (1∶200, Invitrogen, Carlsbad, CA), then washed with PBS.

Intact hearts were frozen in OCT embedding compound (Tissue Tek, Torrance, CA), and 10 µm cryosections were prepared. Slides were fixed and blocked as described above; then incubated with anti-wheat germ agglutinin-Alexa Fluor 488 conjugate antibody (1∶100, Molecular Probes, Eugene, OR), and washed in PBS.

For both, slides were mounted with Vectashield with DAPI (Vector Laboratories, Burlingame, CA) and examined for fluorescence using a Leitz DMRBE fluorescent microscope (Leica, Bannockburn, IL) equipped with a Micro Max digital camera (Princeton Instruments, Trenton, NJ) interfaced with Image Pro Plus software (Media Cybernetics, Bethesda, MD). Three random images were taken for each sample at 40X. The n per condition is reported in each figure legend. Approximately 500–800 cells were counted per condition. Surface area (NCM) or minimal Feret's diameter (cardiac section) were analyzed using the Fovea Pro 4.0 plug-in (Reindeer Graphics, Asheville, NC) for Photoshop Elements 4.0 (Adobe, San Jose, CA). The minimal Feret's diameter is more resistant to artifactual variation in size due to oblique vs. cross-sectional orientation of muscle fibers compared to standard area measurements [57].

### Echocardiography

M-mode echocardiography was performed on mice 3 months after injection under ketamine/xylazine anesthesia using a 15-MHz phased-array probe connected to a Sonos 7500 echocardiographic machine (Philips Medical Imaging, Andover, Massachusetts). In brief, an M-mode cursor was positioned in the parasternal short-axis view perpendicular to the interventricular septum and posterior wall of the LV at the level of the papillary muscles, and M-mode images were obtained for measurement of LV end-diastolic and end-systolic dimension (LVDd and LVDs). The percentage of fractional shortening (%FS) was calculated from the equation %FS  =  [(LVDd – LVDs)/LVDd] ×100. The end diastolic and end systolic volumes, ejection fraction, cardiac output and stroke volume were calculated using the Teicholtz formulas [58]. The same sonographer performed all the studies and resulting calculations and was blinded to the treatment groups of the mice.

### Statistics

Mean values from each experimental group were compared using the two-tailed Student's t test or one-way ANOVA with Student Newman-Keuls post-hoc analysis.
